# Fluid responsiveness predicted by transcutaneous partial pressure of oxygen in critically ill patients

**DOI:** 10.1186/2197-425X-3-S1-A239

**Published:** 2015-10-01

**Authors:** J Xu, Y Yang, H Qiu

**Affiliations:** Intensive Care Unit, Southeast University, Zhongda Hospital, Nanjing, China; Southeast University, Zhongda Hospital, Nanjing, China

## Objectives

Our goal was to study the feasibility of predicting fluid responsiveness by transcutaneous partial pressure of oxygen (Ptc02) in the critically ill patients.

## Methods

This was a single centre prospective study conducted in the intensive care unit of a tertiary care teaching hospital. Patients for whom the attending physician decided to perform a fluid challenge or presence of at least one clinical sign of inadequate tissue perfusion in the absence of contraindication for fluid infusion were eligible to participate in the study. Ptc02 was used to continuously record at baseline, during a passive leg raising (PLR), and then during a 250 ml rapid saline infusion in 10 minutes. Fluid responsiveness is defined as a change of stroke volume≥10% after 250 ml volume infusion.

## Results

Twenty-three patients were included; of whom, 9 responded to volume expansion. In the 9 responders, heart rate, mean arterial pressure, pulse pressure, central venous pressure, cardiac output, stroke volume, Ptc02 all increased significantly (p < 0.05). Fluid responsiveness was predicted by the PLR-induced change of 13.9% in Ptc02 (area under receiver-operating characteristic curve 0.932) with a sensitivity of 77.8% and a specificity of 100%.

## Conclusions

In this prospective study, it is suggested that the newly defined parameter, Ptc02 changes during the volume expansion or PLR appears to be a good parameter to predict fluid responsiveness.

## Trial Registration

NCT02083757

